# The effect of cognitive behavior therapy on anxiety and depression during COVID-19 pandemic: a systematic review and meta-analysis

**DOI:** 10.1186/s12991-022-00417-y

**Published:** 2022-10-09

**Authors:** Nasrin Zamiri-Miandoab, Robab Hassanzade, Mojgan Mirghafourvand

**Affiliations:** 1 Student Research Committee, School of Nursing and Midwifery, Tabriz University of Medical Science, Tabriz, Iran; 2 Department of Midwifery, Bonab Branch, Islamic Azad University, Bonab, Iran; 3 Social Determinants of Health Research Center, Faculty of Nursing and Midwifery, Tabriz University of Medical Sciences, Tabriz, Iran

**Keywords:** Cognitive behavior therapy, Anxiety, Depression, COVID-19

## Abstract

**Background:**

The global spread of coronavirus has caused many physical and mental health problems throughout the world. Depression and anxiety are among the issues that people are experiencing abundantly, along with other mental health disorders, during this period. Cognitive behavior therapy (CBT) is one of the approaches that is effective on improving most of the psychological issues including depression and anxiety. The objective of this systematic review and meta-analysis was to assess the effects of CBT on depression and anxiety during COVID-19 pandemic period.

**Methods:**

English databases such as Cochrane, PubMed, Google Scholar, Web of Science and Persian databases such as SID, MagIran and IranDoc were searched with a time limit of 2019 to 2022. Two researchers independently evaluated the quality of the entered studies based on Cochrane handbook. Subgroup analysis was conducted separately on the basis of being infected with coronavirus, not being infected with coronavirus, and having a history of depression or anxiety before the intervention and internet-based CBT for depression and anxiety. Meta-analysis results were reported using standardized mean difference (SMD) and 95% confidence interval (95% CI). Heterogeneity of studies was analyzed by means of *I*^2^ index; and in the case of heterogeneity presence, random effects model was used instead of fixed effects model. Grading of Recommendations Assessment, Development and Evaluation (GRADE) was used for evaluating the quality of evidence.

**Results:**

Totally, 2015 articles were analyzed of which 11 articles entered meta-analysis. The overall results of meta-analysis showed that mean score of anxiety in the group receiving CBT was significantly lower than the control group (SMD: − 0.95, 95% CI − 1.29 to − 0.62; *P* < 0.00001, *I*^2^ = 94%). In addition, mean score of depression in the intervention group was significantly lower than the control group (SMD: − 0.58; 95% CI − 1.00 to − 0.16, *P* < 0.00001, *I*^2^ = 94%). In addition, the results of subgroup meta-analysis showed that internet-based CBT was effective in reducing of depression (SMD − 0.35; 95% CI − 0.50 to − 0.20; *P* < 0.00001; *I*^2^ = 0%) and anxiety (SMD − 0.90; 95%CI − 1.47 to − 0.33; *P* = 0.002; *I*^2^ = 94%). The evidence about the effectiveness of CBT on depression and anxiety compared with control group on the basis of GRADE approach had low quality.

**Conclusions:**

Meta-analysis results showed that CBT reduced the mean scores of anxiety and depression significantly during COVID-19 pandemic period. Due to the low quality of evidence, conducting more randomized controlled trials with rigorous design is suggested.

*Prospero registration* This systematic review has been registered in Prospero (ID: CRD42021277213).

## Introduction

The worldwide outbreak of coronavirus pandemic since 2019 became a very serious crisis for all people and posed many challenges for world health community as well as research and medical committees [1]. Presently, people from all age groups including children to adults and people with any health background are at risk of coronavirus infection, so that by August 25, 2021, more than 214,000,000 people in the world were infected with the virus of whom more than 4,470,000 people lost their lives [2].

Although a lot of attention is paid to coronavirus pandemic, and its news and information are broadcasted moment by moment, the attention is just paid to the pathogenic aspect of coronavirus, while its psychological effects on society are neglected most of the time [3]. Each traumatic event may reduce people’s sense of security and affect their mental health negatively. Being exposed to coronavirus news and unanswered questions about when the pandemic ends and what its treatments are, along with decreased social relations and widespread quarantines all have negative effects on people’s mental health [3, 4]. Moreover, decline of some people’s income because of widespread lockdowns disrupts families’ well-being and affects their quality of life and consequently their mental health negatively [5]. Extremely rapid transmission and high mortality rate of this disease lead to psychological problems or worsen the former problems in people [6]. Some symptoms such as anxiety, depression, fear, stress, and insomnia are very common during coronavirus pandemic [3].

Depression is a common disorder that presently affects nearly 120 million people all over the world and its prevalence rate is between 10% and 15% in a lifetime according to epidemiological surveys [7]. Anxiety is also another common mental disorder. Mental disorders, according to WHO, account for 30% of nonfatal illnesses and it is estimated that 1 in 5 persons are exposed to anxiety and depression in critical conditions [8]. A study conducted in China showed that prevalence of depression and anxiety increased significantly among general population during COVID-19 crisis [9].

Since COVID-19 pandemic has a lot of implications for personal, social, and emotional health; emotional and social needs of people during this period should receive enough attention from providers of these services and health policy makers, in addition to medical services [10]. Cognitive behavior therapy (CBT) is a psychological therapeutic approach that is extensively used both as a prevention and a treatment method for both medical staff and general population [11]. CBT is a set of methods including cognitive restructuring and behavioral change that helps people control their stress, change their negative behavior and attitudes and, likewise, reduces the symptoms of distress and mental health problems [12].

CBT is an intervention that improves the coping skills in anxiety. This intervention help an individual how respond to stressful events in their life [13]. For mild to moderate depression, CBT even competes with antidepressant medications and a combination of CBT and antidepressant medications increases the effectiveness of treatment. Among all the psychotherapies, CBT is considered the first line of treatment for depression [14]. Thus, effectiveness and efficiency of this treatment are approved for both anxiety and depression.

Considering the increasing cases of psychological problems including anxiety and depression during the recent pandemic and the existence of various psychological therapies, we decided to meta-analysis and systematically review the effectiveness of CBT on anxiety and depression during this period.

## Methods

### Inclusion criteria

The studies entered in this research consist of clinical trials analyzing the effects of CBT on depression and anxiety during COVID-19 pandemic period. Other studies such as editorial studies, case studies, protocol studies, articles with insufficient data and studies with lack of control group were excluded from the present study. In addition, we included the studies just conducted during the COVID-19 pandemic. We excluded the studies which used another intervention for control groups. In this study, the researchers using the keywords they looked up in MeSH such as COVID-19, depression, anxiety and CBT launched their study by searching English databases such as PubMed, Web of Science, Google Scholar, Cochrane as well as Persian databases such as SID, MagIran and IranDoc with a time limit of 2019 to 2022. Moreover, clinical registries such as EU-CTR and IRCT were searched for relevant unpublished studies. We updated our search on 17/08/2022 for last time.

Defined PICO for this study included participants (people during COVID-19 pandemic regardless of sex and age), intervention (CBT), control group (with no intervention) and outcomes (anxiety and depression).

One of the strategies used in this study to search in PubMed database is as follows:

- ((depression [MESH Terms]) OR anxiety [MESH Terms]) AND cognitive behavior therapy [MESH Terms] at COVID-19

### Quality assessment and data extraction

Two researchers (NZ and RH) independently investigated the titles and abstracts of the studies that were obtained via the search for a review study; if obtained data were not sufficient for decision making, the whole article was analyzed and any uncertainty about its suitability for being entered in the study was resolved through consulting with a third researcher (MM).

The researchers extracted the data from the relevant studies on the basis of Cochrane handbook including: first author’s name, year of publication, country, characteristics and design of intervention, age of participants, sample size, type of intervention, type of control group, measuring tools, results and consequences, and then presented the data in Table 1.

**Table 1:** Characteristics of included studies

Author (year)	Design	Country	Sample size	Age	Intervention	Comparison	Outcomes	Outcome measures	Results
Liu (2021)	RCT	China	273	No age limitation	CBT	TAS	Anxiety and depression	HAMD HAMA SDS	CBT was effective
Li (2020)	RCT	China	94	No age limitation	CBT	No counseling	Anxiety and depression	DASS-21	CBT was effective
Heyrat (2020)	Quasi-experimental	Iran	30	Adolescent girls	CBT	No counseling	Anxiety and depression	Beck	CBT was effective
Song (2021)	Clinical trial	China	129	18 years above	CBT	No counseling	Anxiety and depression	PHQ-9 GAD-7 Anxiety self-rating COVID-19	CBT was effective
Shabahang (2020)	RCT	Iran	150	18–30-year-old people	CBT	No counseling	Anxiety and depression	SHAI beck	CBT was effective
Shabahang (2021)	RCT	Iran	152	18–40-year-old people	CBT	No counseling	Anxiety and depression	CVAQ SHAI ASI-3	CBT was effective
Egan (2021)	Randomize trial	Australia and UK	225	18 years above participants	CBT	No counseling	Anxiety and depression	GAD-7 PHQ-9	CBT was effective
Aminoff (2021)	RCT	Sweden	52	18 years above participants	CBT	No counseling	Anxiety and depression	BDI-II PHQ GAD-7	CBT was effective
Wahlund (2020)	RCT	Sweden	670	Adults with no time limitation	CBT	No counseling	Depression and anxiety	GAD-7 MADRS-S	CBT was effective
Dinarvand (2022)	Pilot study	Iran	30	18–30-year-old people	CBT	NO counseling	Anxiety	GAD-7	CBT was effective
Ahmed Ali (2021)	RCT	Saudi Arabia	54	20 to 54-year-old people	CBT	No counseling	Anxiety	COVID-19 anxiety scale	CBT was effective

*HAMD* Hamilton depression scale, *HAMA* Hamilton anxiety scale, *SDS* Self-relating depression scale, *DASS*-21 depression anxiety stress scale, *PHQ-9* patient health questionnaire, *GAD-7* generalized anxiety disorder, *SHAI* short health anxiety inventory, *CVAQ* COVID-19 anxiety questionnaire, *ASI* anxiety sensitivity inventory, *BDI-II* beck depression inventory-II, *MADRS*-*S* Montgomery-Asberg depression rating scale

Two researchers (NZ and RH) independently assessed risk of biases and again evaluated and resolved any inconsistencies via counseling with a third researcher (MM). The studies included were analyzed on the basis of Cochrane Handbook and in regard to six domains of bias (random allocation, allocation concealment, blinding of participant and personnel, blinding of data assessor, incomplete outcome data, and selective reporting) in three levels of low-risk, unclear, and high-risk and the results are presented in Table 2. The quality of evidence was analyzed using Grading of Recommendations Assessment, Development and Evaluation (GRADE) approach and is reported in Table 3. Likewise, for assessing publication bias, funnel plot was plotted for 10 studies that evaluated anxiety.

**Table 2:** Risk of bias summary in included studies

Bias	AhmedAli (2021)	Aminoff (2021)	Egan (2021)	Heyrat (2020)	Li (2020)	Liu (2021)	Shabahang (2020)	Shabahang (2021)	Song (2021)	Dinarvand (2022)	Wahlund (2020)
Random sequence generation (selection bias)	?	Y	?	N	?	?	?	?	?	N	?
Allocation concealment (selection bias)	?	Y	Y	?	Y	Y	N	Y	N	N	Y
Blinding of participants and personnel (performance bias)	N	N	N	N	N	N	N	N	N	N	N
Blinding of outcome assessment (detection bias)	N	N	N	N	N	N	N	N	N	N	Y
Incomplete outcome data (attrition bias)	Y	Y	Y	Y	Y	Y	Y	Y	Y	Y	Y
Selective reporting (reporting bias)	Y	Y	Y	Y	Y	Y	Y	Y	Y	Y	Y
Other bias	Y	Y	Y	Y	Y	Y	Y	Y	Y	Y	Y

Y, low risk; N, high risk; ?, unclear

**Table 3:** Quality assessment of included studies according Grade approach

Quality assessment											MD (95% CI)^a^	Certainty
No. of studies	Design	Risk of bias		Inconsistency		Indirectness		Imprecision		Publication bias	CBT	Certainty
CBT versus control for anxiety												
11	Randomized trials		Serious risk of bias^a^		Serious inconsistency^b^		No serious indirectness		No serious imprecision	Undetected	− 0.95 (− 1.29 to − 0.62)	Low ⨁OOO
CBT versus control for depression												
6	Randomized trials		Serious risk of bias^a^		Very Serious Inconsistency^b^		No serious indirectness		Serious imprecision	Undetected	− 0.58 (− 1.00 to − 0.16)	Very low ⨁OOO

^a^Mean difference (95% confidence interval)

^b^In most of the studies randomization method have not been reported. No blinding have been used due to kind of intervention

^c^Substantial inconsistency (*I*^2^ = 94%)

### Data analysis

The Review Manger (RevMan) software of version 5.3 and Stata version 14.2 software (Stata crop, College Station, TX, USA) were used for meta-analysis and plotting bias forms. Because of the different tools that were used for measuring anxiety and depression, meta-analysis results were reported using standardized mean difference (SMD) and 95% confidence interval (95% CI). Heterogeneity of studies was analyzed by means of *I*^2^ index, and in cases with high heterogeneity, random effects model was used instead of fixed effects model. Subgroup analysis was conducted on the basis of being infected with coronavirus, not being infected with coronavirus, having a history of depression and anxiety before the intervention and internet-based CBT for depression and anxiety. Since the number of included studies in the meta-analysis of anxiety was more than 10 studies, publication bias was assessed with Egger’s test and funnel plot. A meta-regression analysis was performed to estimate the influence of the participants’ type (being infected with coronavirus, not being infected with coronavirus, having a history of depression and anxiety) on heterogeneity.

## Results

**Fig. 1:**
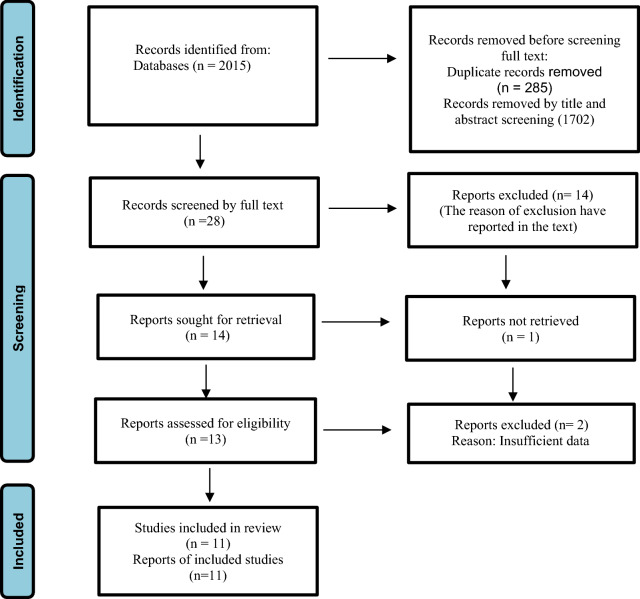
PRISMA flow diagram (2020) of screening, selection process and inclusion study

A total of 2015 articles were obtained from the search in different databases of which 1987 articles were excluded due to duplicates or the irrelevancy of their titles or after reviewing their abstracts. Of 28 remaining articles, 14 of the articles did not enter the systematic review due to the following reasons: Mohammadi et al. [15] and Norred et al. [16] articles were excluded from the study because of its editorial type. Hosseinzade et al. [17] article did not include to the study, because it was a study protocol. Kalvin et al. study [18] was a commentary study. Likewise, Mahoney et al. study [19], Zhang et al. study [20], Araghi et al. study [21] and Uysal et al. study [22], Green et al. study [23] were excluded from the study due to lack of the control group. Johnco et al. study [24] and Sharrock et al. study [25] did not enter the study, because the studies were conducted previously and the follow-up was conducted again in the coronavirus period. Fadhli et al. study [26] was excluded, since the mean and standard deviation of results before and after the intervention were reported as a single value together and the effects of the intervention were not properly stated. Likewise, Perri et al. study [27] and Hamed et al. study [28] were excluded from the study, because its control group had received another type of intervention. Of the 14 remaining articles, one article was not retrieved, because the full text of Barve et. al [29] study was not available, we emailed the authors but we did not receive any response; so this article was not included in the present study. In addition, two of the articles including Oehler et al. study [30] due to lack of anxiety scores before and after the intervention and LV et al. study [31] because of presenting insufficient data were excluded from the study. Finally, 11 articles were entered into the study (Fig. 1).

**Fig. 2:**
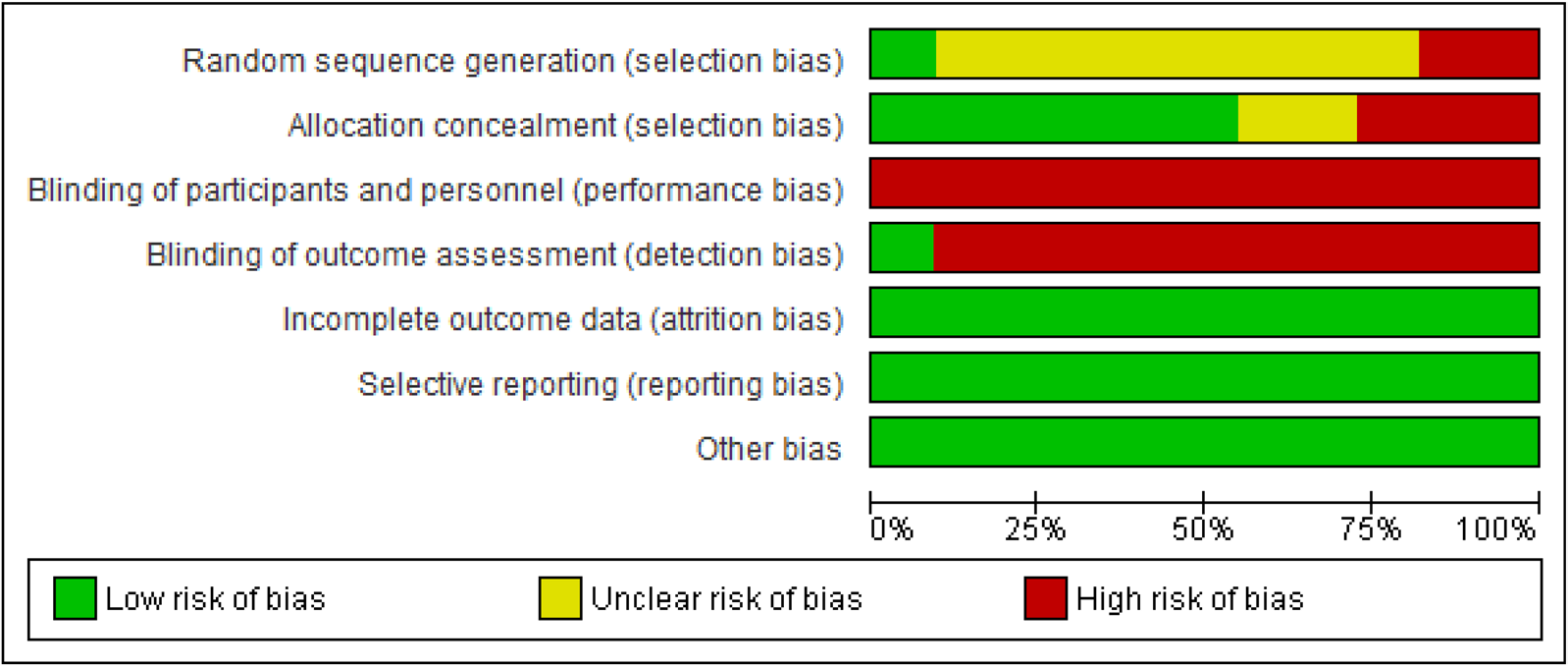
Risk of bias graph: review authors judgments about each risk of bias item presented as percentages across all included studies

**Fig. 3:**
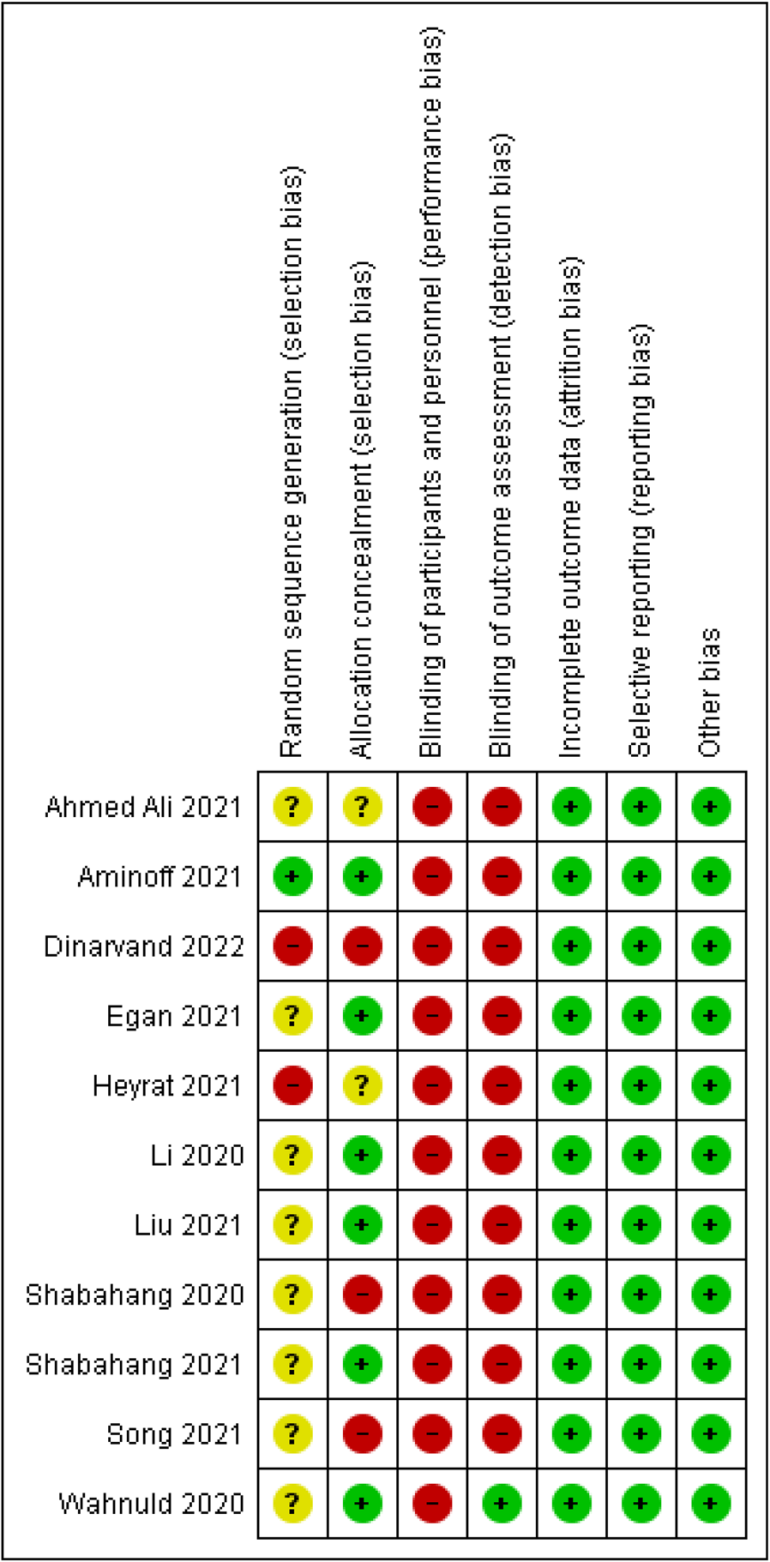
Risk of bias summary: review authors judgment about each risk of bias item for each included study

The risks of biases of the entered studies are presented in Table 2. Most of the studies did not provide sufficient data on how randomization was done and so are placed at unclear risk level; likewise, most of the studies are put at low risk level regarding allocation concealment bias. All of the studies are high risk in terms of performance bias. All studies except one [32] are at high-risk level in terms of detection bias. Moreover, all studies are low risk in terms of reporting bias and attrition bias (Figs. 2 and 3).

### The specifications of included studies are as follows

Three of the studies were conducted in China [1, 33, 34], four of them in Iran [35–38], one of them was the common study between Australia and United Kingdom [39], two of them in Sweden [32, 40] and one of them in Saudi Arabia [41]. All of the studies were clinical trial but one of them was a pilot study [38]. All of the studies were conducted during the COVID-19 pandemic. The largest and smallest sample sizes were 670 [32] and 30 [35, 38]. Five studies were conducted face to face [1, 34–36, 38] but the others were internet-based. In 4 studies, there was no information about the gender of participants [33, 36–38]. One study was conducted just on girls [35] and others were on both gender. Most of the studies were conducted on adults above 18 years, just in one of the studies, participants were adolescents [35] and two of them had no age limitation [1, 34]. The scales used in the studies were different such as Hamilton Depression Scale (HAMD), Hamilton Anxiety Scale (HAMA), Self-related Depression Scale (SDS), Depression Anxiety Stress Scale (DASS-21), Patient Health Questionnaire (PHQ-9), Generalized Anxiety Depression (GAD-7), Short Health Anxiety Inventory (SHAI), COVID-19 Anxiety Questionnaire (CVAQ), Anxiety Sensitivity Inventory (ASI), Beck Depression Inventory (BDI), and Montgomery Asberg Depression Rating Scale (MADRS-S). Most of the articles examined both outcomes (depression and anxiety) but two of them examined just anxiety [38, 41].

### Meta-analysis

Random-effects meta-analysis on 6 studies and 1269 participants showed that the mean score of depression in the intervention group was significantly lower than the control group (SMD − 0.58; 95% CI − 1.00 to − 0.16; *P* < 0.00001) and heterogeneity level of *I*^2^ = 94% were obtained. Subgroup analysis results on the basis of being or not being infected with coronavirus or having a history of depression were as follows.

**Fig. 4:**
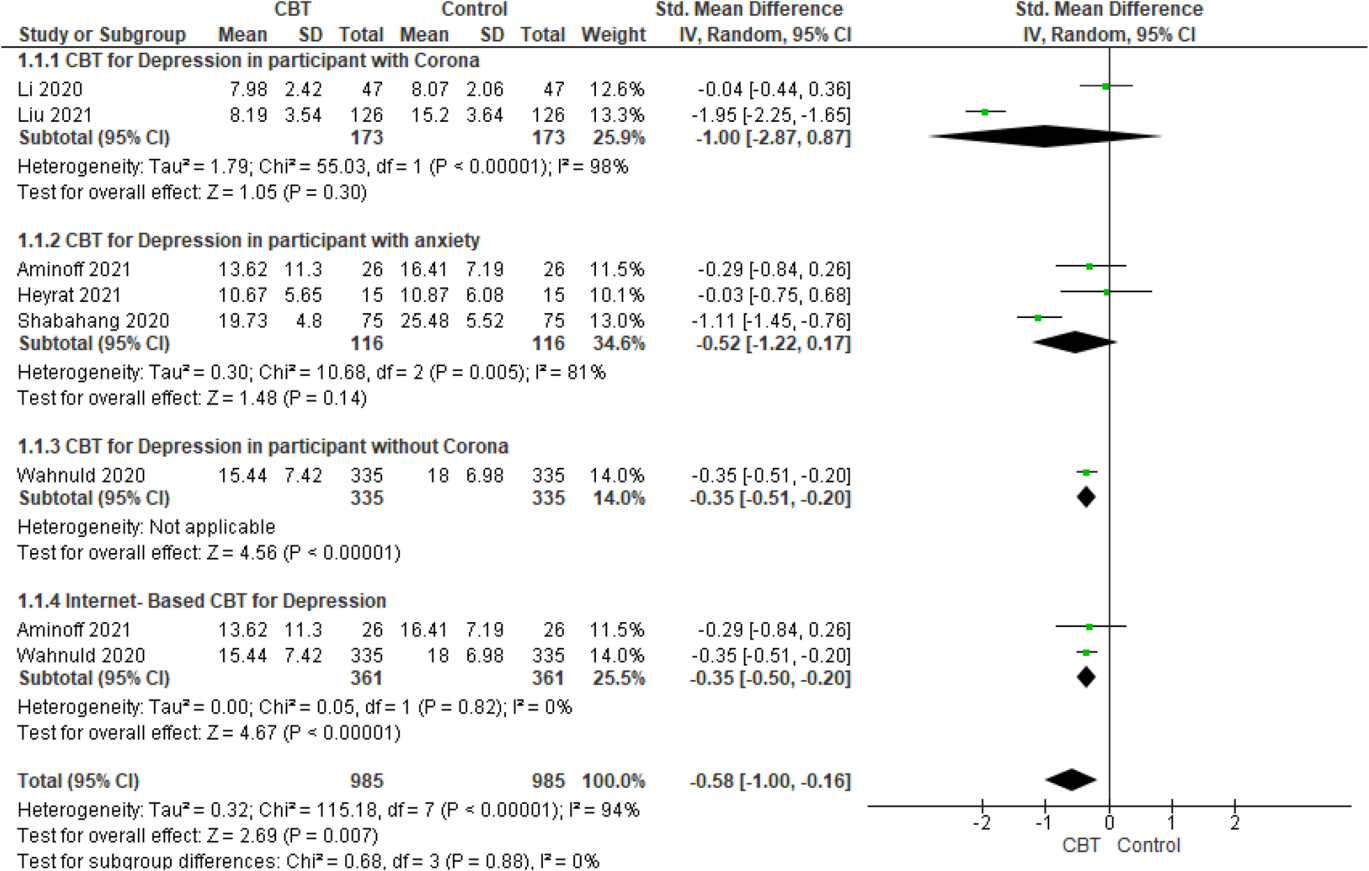
Meta-analysis of effect of CBT on depression during COVID-19 pandemic

In terms of depression variable, just one study was performed on people not infected with coronavirus and meta-analysis results showed that CBT had a positive effect on lowering the mean score of depression among the patients not infected with coronavirus (MD − 0.36; 95% CI − 0.51 to − 0.21; *P* < 0.00001). However, there was no statistically significant difference between two groups for those infected with coronavirus (SMD − 1.00; 95% CI − 2.87 to 0.87; *P* < 0.00001; *I*^2^ = 98%) and people with a history of depression (SMD − 0.52; 95% CI − 1.22 to 0.17; *P* = 0.005; *I*^2^ = 81%). There were two studies that implemented CBT via the internet for depression. Subgroup analysis was conducted on these studies. The results of the meta-analysis showed that internet-based CBT was effective in reducing of depression (SMD − 0.35; 95% CI − 0.50 to − 0.20; *P* < 0.00001; *I*^2^ = 0%) (Fig. 4).

Random-effects meta-analysis on 11 studies and 1859 participants showed that the mean score of anxiety in the intervention group was significantly lower than the control group (SMD − 0.95; 95% CI − 1.29 to − 0.62; *P* < 0.00001; *I*^2^ = 94%). Subgroup analysis results on the basis of being or not being infected with coronavirus or having a history of anxiety were as follows.

**Fig. 5:**
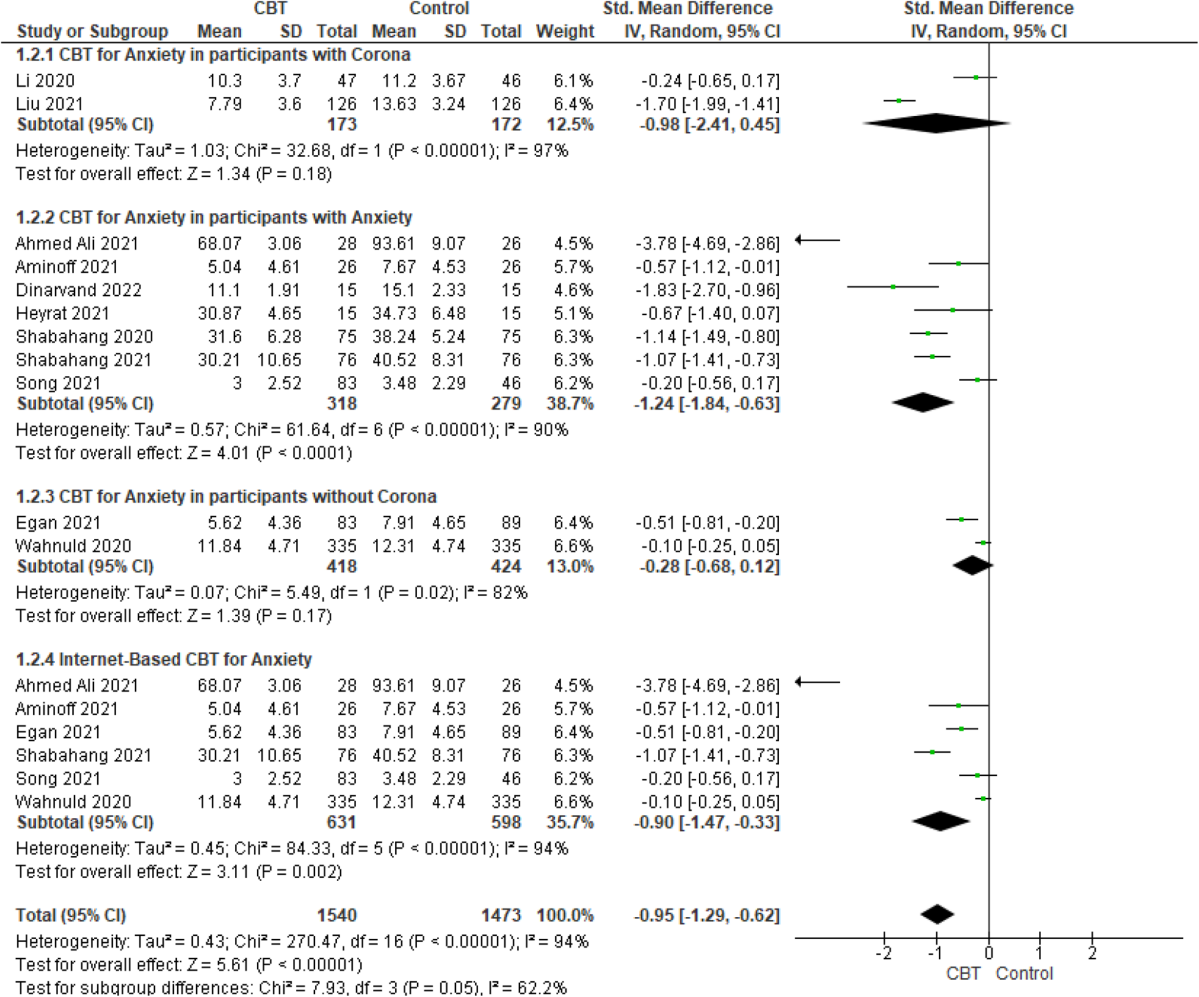
Meta-analysis of effect of CBT on anxiety during COVID-19 pandemic

In terms of anxiety variable, subgroup analysis results showed that CBT had significant effect just on lowering anxiety score for those people with a history of anxiety (SMD − 1.24; 95% CI − 1.84 to − 0.63; *P* < 0.00001, *I*^2^ = 90%) but for people infected (SMD − 0.98; 95% CI − 2.41 to 0.45; *P* = 0.18, *I*^2^ = 97%) and not infected with coronavirus (SMD − 0.28; 95% CI − 0.68 to 0.12; *P* = 0.17; *I*^2^ = 82%) there were no statistically significant difference between the intervention and control groups. There were 6 studies that assessed the effect of internet-based CBT on anxiety. Subgroup analysis was conducted on these studies. The meta-analysis results showed that internet-based CBT had a significant effect on anxiety (SMD − 0.90; 95% CI − 1.47 to − 0.33; *P* = 0.002; *I*^2^ = 94%) (Fig. 5).

**Fig. 6:**
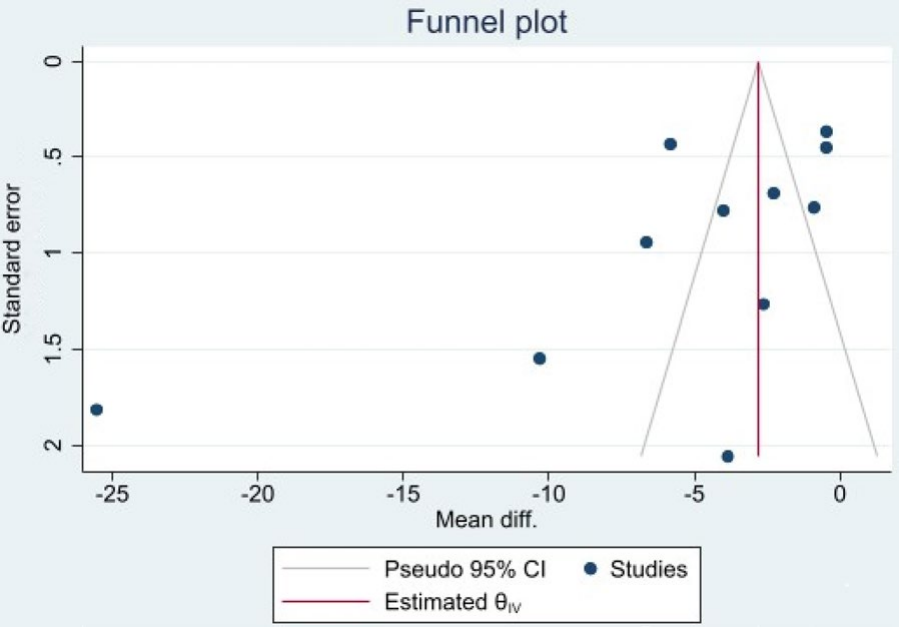
Funnel plot of the effect of CBT on anxiety during COVID-19 pandemic

The asymmetrical appearance of the funnel plot indicates the probable presence of publication bias in the anxiety-related studies (Fig. 6). This was confirmed by the results of an Egger regression that showed significant publication bias (*P* = 0.024).

The results of meta-regression model indicated that in both variables of anxiety and depression, none of the two groups (participants with corona and participants with anxiety) did not show significant difference compared to the participants without corona.

The evidence about the effectiveness of CBT on depression and anxiety compared with control group on the basis of GRADE approach had low quality, so the results were close to reality with low reliability. The results of evaluation of evidence utilizing GRADE are shown in Table 3.

## Discussion

According to most of the studies, COVID-19 pandemic has a negative impact on individual mental health so different psychological approaches should have been used to improve society’s well-being. CBT is one of the effective approaches for psychological disorders. In the present study, the effects of CBT on people’s depression and anxiety in COVID-19 pandemic period were analyzed. Based on the overall results of meta-analysis, CBT had a significant effect on reducing anxiety and depression. Since we did not find systematic review studies on the effects of CBT on anxiety and depression in coronavirus period, we reviewed similar studies. No study was found that examined both outcomes so we have reviewed outcomes separately. There were some studies that evaluated the effect of CBT on the anxiety.

Hall et al. [42] evaluated the effects of CBT on the anxiety disorder. Meta-analysis results in the study were similar to the present study and showed that CBT was significantly effective on reducing anxiety in the intervention group. Unlike the present study, in the Hall et al. study, control groups have received different interventions, such as supportive therapy, acceptance and commitment therapy, discussion group and etc. In a systematic review and meta-analysis, Sigurvinsdóttir et al. [43] studied the effects of CBT on anxiety. Meta-analysis results showed that CBT was significantly effective. Sigurvinsdóttir study was conducted just on children and adolescents, and so its results could not be generalized to other age groups but the present study has no age limitation in inclusion criteria.

There are a number of articles that evaluated the effect of CBT on depression. In a systematic review, Lopez-Lopez et al. [44] investigated the effects of CBT on depression. This study conducted just on adults above 18. In the present study, we have no age limitation. In a systematic review and meta-analysis, Santoft et al. [45] studied the effect of CBT on depression. This study has reviewed more articles than the present study (34 articles) and control groups have received different interventions, such as placebo pill, psychotherapy and etc. The results of Santoft et al. study similar to the results of the present study showed that CBT significantly reduced symptoms of depression in the intervention group. All of these studies were conducted before COVID-19 pandemic.

CBT is a therapeutic approach that is suggested for the treatment of depression and anxiety by Aaron beck. CBT helps the patients change their thoughts and through which leads to better feelings in them [46]. Since negative thoughts have a detrimental influence on developing emotional disorders such as anxiety and depression, CBT alleviates anxiety and depression symptoms through recognizing, challenging, and changing negative thoughts and replacing them with logical thoughts [47]. Moreover, CBT improves many mental health disorders such as anxiety and depression through developing capabilities necessary for coping with everyday life events [48], and since many studies approve that CBT is one of the valid therapeutic methods, it is extensively used. Most of the studies detected changes in the brain (such as prefrontal hyperactivity) during psychological disorders, such as anxiety and depression. Findings of studies showed that the abnormal hyperactivities of the prefrontal decrease following treatment. In addition, results showed that CBT can enhance the prefrontal control structures [49, 50]. In some of the studies, researchers used internet-based CBT instead of face to face CBT due to COVID-19 pandemic.

The results of the subgroup meta-analysis showed that ICBT (internet-based CBT) is effective in reducing depression and anxiety. In the Farrer et al. study [51], ICBT was effective in reducing depression, although they used weekly telephone tracking too. In Herman et al. study [52], ICBT was effective in reducing anxiety. They followed up the participants for 5 years. The results of Etzelmueller et al. [53] and Lau et al. [54] studies were similar to the present study too and ICBT was effective in reducing depression and anxiety. However, Etzelmueller et al. study has reviewed more articles than the present study (19 articles) and they included adults and older adolescents in their study. In the present study, we have no age limit. In addition, the participants of the Lau et al. study were postpartum women. All of these studies were conducted before COVID-19 pandemic.

## Strength and limitation

This systematic review and meta-analysis has a number of weaknesses and strengths. One of the limitations was the small sample size of the included studies. In addition, we had just 11 studies which also limited our sub-group analyses. The other limitations of the present study were different study designs, participant’s ages and outcome measures. In addition, the most of the included studies did not clearly stated their methods of randomization and so were placed in unclear risk level in terms of randomization bias, just one study [39] was low risk in this regard. In none of the studies blinding was performed, however, in just one study [31], analyzer’s blinding was done. Of other weaknesses of the included studies was their different inclusion criteria, for instance, some of the studies were conducted on people infected with coronavirus, some on people having a history of depression and anxiety, and some on people not infected with coronavirus. Likewise, in most of the studies duration of follow-up was not stated. In most of the studies, because of coronavirus pandemic and the necessity of quarantine and observing social distance, CBT counseling sessions were performed online and via internet that is one of the weaknesses of these studies, because there was no possibility of face to face meetings between counselors and clients; however, this was the best decision in the pandemic period and provided people with the opportunity to benefit from counseling and the results also showed the effectiveness of the method.

One the strengths of the present study is that all of the included studies are at low-risk level in terms of reporting bias and attrition bias. Likewise, in the present systematic review, all age groups regardless of their sex entered the study, so the results are generalizable to all age groups and both sexes. Of other strengths of this study, we can refer to subgroup analysis.

## Conclusions

Meta-analysis shows that CBT is significantly effective on reducing depression and anxiety scores in COVID-19 pandemic period. Therefore, extensive use of this method during coronavirus pandemic and other similar pandemics is recommended. It is suggested that the effectiveness of other psychological approaches on the problems developed during the pandemic to be analyzed in other studies. Moreover, since the need for counseling and psychological interventions is inevitable due to psychological and mental disorders people experience during different kinds of crises such as natural or social crises or pandemics etc., it is recommended that governments and communities put paying enough attention to these methods on their agenda.

## Data Availability

All data generated or analyzed during this study are included in this published article.

## Abbreviations

CBT: Cognitive behavior therapy
SMD: Standardized mean difference
MeSH: Medical subject heading
EU-CTR: European Union Clinical Trials Register
IRCT: Iranian Registry of Clinical Trial
CI: Confidence interval
WHO: World Health Organization
RCT: Randomized controlled trial
GRADE: Grading of recommendations assessment, development and evaluation
HAMD: Hamilton depression scale
HAMA: Hamilton anxiety scale
SDS: Self-relating depression scale
DASS: Depression anxiety stress scale
PHQ: Patient health questionnaire
GAD: Generalized anxiety depression
SHAI: Short health anxiety inventory
CVAQ: COVID-19 anxiety questionnaire
ASI: Anxiety sensitivity inventory
BDI-II: Beck depression inventory-II
MADRS-S: Montgomery-Asberg depression rating scale
